# Risk factors for upper limb fractures due to unintentional injuries among adolescents: a case control study from Sri Lanka

**DOI:** 10.1186/s12889-022-14154-0

**Published:** 2022-09-26

**Authors:** Hemali Jayasekera, Samitha Siritunga, Upul Senarath, Paramjit Gill

**Affiliations:** 1 National Programme for Prevention and Control of Non-Communicable Diseases, Ministry of Health, Colombo, Sri Lanka; 2 Department of Community Medicine, Faculty of Medicine, University of Colombo, Colombo, Sri Lanka; 3 Division of Medical Sciences, Warwick Medical School, University of Warwick, Coventry, UK

**Keywords:** Adolescents, Risk factors, Injuries, Upper limb factures

## Abstract

**Background:**

Injuries are the number one cause for morbidity and mortality among adolescents. Adolescent fractures are a hidden public health problem in Sri Lanka. Upper limb fractures are common in adolescents due to various risk factors. Many injuries are predictable and can be prevented by identifying the risk factors. The aim of the study was to determine the risk factors for upper limb fractures among adolescents in Sri Lanka.

**Methods:**

A case control study was undertaken with 450 cases and 450 controls. Cases were recruited consecutively from all major hospitals among the adolescent victims who had admitted with newly diagnosed upper limb fractures in the district of Colombo. Controls were apparently healthy adolescents from the same district and excluded who had previous upper limb fractures. The age and gender were not matched in selecting controls since these two factors were potential risk factors for adolescent fractures according to previous literature. Risk factors for upper limb fractures were assessed by odds ratio (OR) with 95% confidence interval (CI) and adjusted for possible confounding by performing logistic regression analysis.

**Results:**

The mean age of the cases was 13.62 years with a Standard Deviation (SD) of 2.8 and controls was 12.75 years (SD = 2.7) respectively. Having a high standard of living index (OR = 3.52; 95%CI: 2.3–5.2, *p* < 0.001), being in a high social class category (social class I & II) (OR = 2.58, 95%CI: 1.7–3.92, *p* < 0.001), engage in physical or sports activity (OR = 9.36; 95%CI: 3.31–26.47, *p* < 0.001), watching television (OR = 1.95; 95%CI: 1.18 -3.22, *p* = 0.009), playing video or computer games (OR = 2.35; 95%CI: 1.7–3.24, *p* < 0.001), and attending extra classes (OR = 1.82; 95%CI: 1.2–2.7, *p* = 0.007) were risk factors for having a upper limb fracture.

Risk factors for upper limb fractures following adjusted for confounders were siblings in the family (aOR = 11.62, 95% CI: 6.95–41.29, *p* = 0.03) and attend extra classes after school hours (aOR = 2.51, 95%CI: 0.68–0.93, *p* = 0.04). Two significant effect modifications between being a Buddhist and low standard of living index (*p* < 0.001) and having one sibling in the family and attend extra classes after school hours (*p* = 0.01) were observed.

**Conclusions:**

Modifiable risk factors in relation to lifestyle factors and socioeconomic position were important determinants of upper limb fracture risk in adolescents. Many fractures can be prevented by strengthening awareness programmes in the community.

## Background

Injuries are the number one cause of morbidity and mortality among adolescents. Injuries can be commonly classified as intentional or unintentional injuries. Road traffic accidents, poisoning and suffocation, falls, drowning, electrocution, animal attacks, explosions, burns, natural disasters and other accidental injuries are some examples of unintentional injuris. Intentional injuries are interpersonal violence involving family or community, and those that are self directed, such as suicide or self harm or war injuries [1]. Unintentional injuries have a significant impact on childhood disabilities and deaths in the world [2].

Fractures have been identified as a major consequence of injuries and fractures of the limbs significantly limit their functional capacity. It can lead to reduced productivity and quality of life of adolescents. They belong to economically productive age group in a country as such prevention of injuries among adolescents will be an investment for a developing country [3]. Adolescents have to live in a world with potential hahzards as adults design and produce products for their own use [2].

Fractures are common public health problems among children and adolescents all over the world [3]. According to the WHO, the overall fracture rate was 32.4% of the unintentional injuries among children under 15 years of age [4].

Upper limb fractures caused by injuries account for 80% of all fractures. This has been contributed to a significant level of morbidity and mortality [5]. The literature has revealed that the types of fractures depend on the magnitude and direction of the force place on a bone [6]**.** At different ages during the growth period, the type of fracture varies due to changes in bone composition [7]. There is an increase in the tendency to sustain a fracture in children and adolescents under the age of 19, although most of these patients are generally healthy [5]**.**

According to previous literature, poor socio-economic status of an adolescent was associated with sustain a fracture [8]. Further, the genetic factors, lack of exercise, obesity, poor nutritional status, and exposure to trauma were the main risk factors involved in sustaining a fracture in this age group [9]. The author also pointed out that fracture rates were gradually increasing among adolescents due to environmental changes which was a result of urbanization in the recent past. In developing countries, preventive measures are not taken in schools or in playground areas for the safety of children due to lack of resources.

The sociodemographic factors are contributed as risk factors for adolescent fractures as in previous literature. A cross sectional study design was carried out in out patient clinics of the Department of Orthopedics and Traumtology in a children’s hospital situated in the southern region of Italy revealed that adolescent males were more prone to fractures than adolescent females (*p* < 0.001) [4]**.** Cohort study design was carried out in Australia among hospitalized patients revealed that the risk of having a fracture increased gradually from the age of 12 to the age of 19 in males and vice versa in females [10]. Further, in a study carried out in Emergency Departments of United States revealed that unintentional injuries were high among males, in people with low socioeconomic status, and among 15 to 19 years age group [11].

The transition period from childhood to adulthood is considered very precious, as children undergo rapid changes in physical, social and psychological development during this period. Adolescents think that they are invulnerable, prefer to be independent and to practice healthy and unhealthy behaviors [3]. A degree of risk-taking behavior is normal in adolescents since most of them prefer to engage in high-risk activities and aggressive behaviors [12]. As a result of urbanization and development of technologies such as smart phones, tablets and computers, adolescents spend less time for outdoor activities. The competition in the education has worsen the situation as most of the adolescents attend extra classes after school hours to get through Advanced Level examinations with best results. The literature stated physical activity helped to reduce the fracture risk in children although there was a small chance of having an injury and it increased the bone mineral density which helped to strengthen the bones in adolescent age [13]. A population-based case control study showed that participation in light physical activity decreased the risk of fractures (OR = 0.8, 95%CI: 0.7–1.0) among adolescents [13]. Further, the children in the United Kingdom engaging in daily vigorous physical activity had double the risk (OR, 2.06; 95% CI:1.21–1.76) of sustaining a fracture [14]. Therefore, adolescents can be encouraged to do light physical activities to improve their health. Further, the study carried out in Tasmania also revealed that time spent on television, computer, and watching videos in both sexes was significant, and there was a 1.6-fold risk of succumbing to wrist and forearm fractures in both sexes (OR = 1.6; 95%CI; 1.1–2.2) [13].

A study done on patients who were treated at orthopedic post-surgical clinics in a tertiary care hospital in Sri Lanka revealed that the commonest cause of sustaining a fracture was a fall. The study had revealed that 35.8% of patients with fractures following injuries were children and adolescents. Upper limb fractures were the commonest type of fractures (83.2%) seen among adolescents in Sri Lanka [15]. Since published data was not available in the local context to identify the risk factors and the burden of upper limb fractures among adolescents, the current study filled this gap in the body of knowledge. Prioritization of strategies with regard to primary prevention will ease the economic burden since Sri Lanka is still a developing country. Many adolescents at this age attend schools during the day time and they engage in sports related activities and other physical activities.

The study aimed at assessing the potential risk factors for upper limb fractures among adolescents to target preventive programmes at field level. Main objective of the present study was to determine the risk factors for upper limb fractures due to unintentional injuries among adolescents aged 10 to 19 years attending selected government hospitals in the district of Colombo, Sri Lanka.

## Methods

An unmatched case control study was performed to determine the risk factors for adolescent’s upper limb fractures with cases recruited consecutively from hospitals and a control group recruited purposively from the community. A control was selected from the community from an updated eligible family register with the assistance of the Public Health Midwife (PHM) of the area. PHM is the grassroot level public health officer providing maternal and child health services in the community. PHM has maintained the eligible family register for her area with the information of adolescents. Therefore, PHM had assisted to recruit a healthy adolescent as a control if a case was resided in a same Grama Niladhari division area. The cases and controls were not matched for age and gender as the magnitude of the effect of potential risk factors namely age and sex had already been assessed as risk factors in the present study. The previous literature covering the local context had determined similar risk factors by carrying out an unmatched case control study [16, 17]. The study was conducted in the district of Colombo, in Sri Lanka among adolescents attending Accident Services Units (ASU) or Primary Care Units (PCU) of six major hospitals in the above district during 2018 to 2019. All major hospitals (six in number) in the district of Colombo were included as study setting.

### Selection of cases

Cases were adolescents aged 10 to 19 years who resided in the district of Colombo for the last one year, and who had been admitted to a tertiary or secondary care hospital in the same district with a newly diagnosed upper limb fracture following an unintentional injury. Adolescents who were in intensive care unit with severe trauma at the time of data collection, adolescents with pathological fractures**,** fractures following epilepsy or due to any medical conditions and adolescents who had existing functional disabilities were excluded from the study with the opinion of experts namely Orthopedic Surgeons and General Surgeons. Cases were identified from the admission registers of the ASU or PCU of the hospitals with the assistance of the above clinical specialists. However, adolescents with upper limb fractures due to road traffic accidents were excluded from the study since they had different set of risk factors as found in previous litrature [18]. The literature revealed that the risk factors for adolescent fractures due to transport injuries were mainly dependent on external causes, for example, factors associated with the driver, pedestrians, vehicle, road conditions and environmental conditions. Adolescents with upper limb fractures fulfilling the eligibility criteria were selected consecutively from the Accident Service Units or Primary Care Units of the above-mentioned hospitals until the required number was obtained.

### Selection of controls

Apparently healthy adolescents who did not have any documentary evidence of an upper limb fracture in the past and resided in the same district more than one year period was defined as controls. Age and sex were not matched with cases as described previously [16, 17]. A control was selected from the same Grama Niladhari division where a case had reported in the district of Colombo. PHM who was a grass root level health care officer in a Medical Officer of Health (MOH) area in Sri Lanka assisted data collectors to select a control using the updated eligible family register. The source population of this study was all children living in the district of Colombo who were at risk of upper limb fractures [18]. Adolescents who were critically ill and adolescents found to have had an upper limb fracture in the past were excluded. A purposive sampling method was used to select a control. The confidentiality of the participant was strictly maintained throughout the study period. The data collection was completed after recruiting 450 eligible controls.

### Sample size calculation

The sample size for cases (*n* = 450) and controls (*n* = 450) for univariate analysis was calculated by ratio of one control per case with 5% significance level, beta error of 0.2 and adding 5% for non-response rate to detect the smallest risk (odds ratio of 1.5 for participation of sports on upper limb fracture in Tasmania [13]. The incidence rate of the risk factor among the community controls in this study was 29% as in the study carried out in Tasmania. The authors could not find any published literature from Sri Lanka to match for the local setting.

### Study variables and validated questionnaires used in the study

The principal investigator (PI) developed a conceptual framework using previous literature to identify potential risk factors during the design stage [19, 20]. An interviewer administered questionnaire was formed with the assistance of expert group to collect data on sociodemographic characteristics, economic characteristics and potential risk factors of upper limb fractures from both cases and control groups. These included factors related to personal (age, sex, ethnicity, level of education, whether living with a parent or caregiver), socio economic status namely monthly income of parents, social class and standard of living, lifestyle and leisure time related activities, social habits including smoking and alcohol consumption, engagement in sports, athletics, physical activities and exercise, predisposing factors including episodes of fasting and previous history of epilepsy [9] and consumption of food including milk. However, some of the potential risk factors were not significant in univariate analysis as such bivariate analysis was performed with factors which were significant. The factors which were not significant as potential risk factors to have an upper limb fracture were social habits namely consumption of alcohol and smoking, predisposing factors such as history of epilepsy and episodes of fasting, food habits such as consumption of meat, fish, milk etc.(*p* > 0.05) with 95% confidence interval.

Father’s or caregiver’s occupation was used to assess the social class of the family as in previous literature [16]. It was a validated and culturally adopted instrument used in the local context [21]. A composite index was developed in the original instrument to measure the social class by considering father’s occupation. Father’s occupation was categorized into leading professions (social class I), lesser professions (social class II), skilled worker (social class III), partly skilled worker (social class IV), unskilled worker (social class V) as mentioned in Table 1.

**Table 1:** List of variables and operational definitions

Term	Definition used in the study
Social class	Social class was determined by father’s occupation according to the following categorization [16, 21]:
	Social class 1—Leading professions (Professional and Managerial)
	Social class 11—Lesser professions (Teacher, nurse)
	Social class 111—Skilled workers and non-manual workers (Armed forces, Police, Clerks, Shop keepers)
	Social class 1 V- Partly skilled workers (Farmer, Estate worker, Skilled laborer)
	Social class V – Unskilled workers—Elementary occupation
	High social class—Combination of social class I and II
	Low social class – Combination of social class III, IV and V
Standard of living index	This was based on the standards related to the demographic & socioeconomic characteristics of a household’s formats described by Ayed et. al., [22] carried out in demographic and health survey comparative studies. It was modified to local setting and used in recent studies [23]. The adolescents’ housing conditions, basic utilities and the ownership of electrical items and vehicles were considered. E.g.: When assessing the ownership of the house, two marks were given if the house is owned by adolescent’s parents or caregiver, one mark was given if the house was rented and zero mark was given if they lived in someone else’s house. The following amenities and utilities were also assessed:
	Availability of electricity and the ownership of number and type of electrical appliances
	Availability of a vehicle and the ownership of number and type of the vehicle
	Type of toilet facilities
	Type of water source
	Material used to build walls and floor of the house
Permanent resident	Adolescent residing for one-year period in the same Grama Niladhari division in the district of Colombo
Adolescent	Age 10–19 years old children
Newly diagnosed	A person who is diagnosed for the first time with documentary evidence and radiological investigation to have upper limb fracture within one week following an injury
Care giver	A person who provides ongoing care and assistance without any payment for a family member or a friend who needs support due to physical, cognitive or mental health condition
Unintentional injury	The events caused without the intention of any person / party/ group or community and those are not inflicted by deliberate means
Major hospitals	All secondary and tertiary care hospitals where specialists in Surgery and Orthopedic Surgery and X ray investigations were available
Road traffic accidents	Any accident occurred due to involvement of a vehicle during any mode of transportation involving land transport accidents such as avenues, streets, roads, highways, express way, air transport accidents, and water transport accidents. The victim may either be the vehicle occupant or others exposed to accident
Physical activity	Any bodily movement produced by skeletal muscles that requires energy expenditure [22] Light activities such as a person can talk and sing and the heart beat slightly faster than normal. Mild activities such as a person can talk but cannot sing and the heart beat faster than normal, heavy intensity is a person cannot talk or his/her talking is broken by large breaths and his/her heart rate increases a lot
Physical exercise	Physical exercise is a subcategory of physical activity that is planned, structured, repetitive and purposeful in the sense that the improvement or maintenance of one or more components of physical fitness is the objective. Physical activity includes exercise as well as other activities which involve bodily movement and are done as part of playing, working, active transportation, house chores and recreational activities [22]
Sport	An activity involving physical exertion, skill and/or hand–eye coordination as the primary focus of the activity with elements of competition where rules and patterns of behavior governing the activity exist formally through organizations
Watching television	Watching television less than 1 h/ 1–2 h/ more than 3 h per day in a week day and in weekends separately
Grama Niladhari Division	An administrative area to carry out the assigned duties of central government at divisional level. A Grama Niladhari (Village Officer) is appointed by the central government to carry out administrative duties

The standard of living index was assessed using a previous validated instrument used in a demographic and health survey [22]. This instrument was modified and validated for the local set up [23]. The questionnaire was developed to assess the housing condition of the adolescent, type of vehicle owned, type of utilities and amenities available in adolescent’s house. Each response was given a score on a previously decided weighted scoring system. The total score obtained by each participant was calculated and they were categorized under high, medium or low standard of living accordingly (Table 1). The range of score to categorize high, medium and low standard of living was decided according to previous literature [23]. The participants who had received the highest score were belong to high standard of living and followed by medium and low standard of living. It was administered as an interviewer administered questionnaire by trained data collectors.

A Global School Based Student’s Health Survey (GSHS) for adolescents was conducted in 2016 in Sri Lanka [19]. The instrument used for that survey was developed by Ministry of Health in Sri Lanka and it was validated and culturally adopted for local context. It was used to develop the questions on lifestyle related factors. A guideline developed by the Ministry of Sports in Sri Lanka was used by authors to develop the questionnaire to assess sports or athletics, physical activities, and exercise related activities in this study. The guideline had been developed to assess the sports related activities or physical activities and sedentary behavior specifically for adolescents to suit for the local context [24].

The judgmental validity was assessed by an expert panel including Orthopedic Surgeons, Pediatric Surgeons, Consultant Community Physicians and General Surgeons. Face validity was ensured by the expert panel who checked for potential risk factors for having an upper limb fracture and assuring the instrument was able to measure all the risk factors. Consensual validity was determined by assessing agreement among experts on the subject on whether or not the instrument was a valid one with which to measure the desired variables [25]. Several consultative meetings were conducted by the principal investigator to assess the judgmental validity of the questionnaire. The instrument was piloted in a different district prior to the main study.

Following the appraisal of validity, a team of pre-intern medical graduates were trained by the principal investigator to collect data from the cases and controls separately. Informed written consent was obtained from the eligible participants and their parents or caregivers before recruiting as study participants. The final instrument used in the study to determine the risk factors for adolescent’s upper limb fracture was a pre-coded interviewer administered questionnaire.

### Measures taken to improve the quality of data

To avoid misclassification, the control group was recruited after interviewing them for previous histories of upper limb fractures. Moreover, they were recruited if there was no documentary evidence to prove that they had sustained previous fractures. To minimize selection bias, the ideal method is to recruit cases and controls from the same setting. However, the controls were not selected from hospitals. The hospital controls came from different socio-economic backgrounds to those of the cases. Instead, a control was selected from the same Grama Niladhari division where the case resided. Conducting interviews at a participant’s residence, maintaining privacy, making a prior appointment to visit the participant’s residence and involving the PHM to identify controls in the community resulted in minimizing the non-response rate among controls. The required sample size for the study was calculated for the smallest odds ratio for potential risk factors following extensive literature review to minimize possible chance errors.

Operational definitions used for each variable in the current study are given in Table 1**.**

## Data analysis

The Statistical Package for Social Sciences (SPSS) version 20.0 was used for data analysis.

The variables identified to assess potential risk factors for upper limb fractures were extracted from the study instruments by the author and entered into the SPSS software. The distribution of cases and controls for sociodemographic factors, socioeconomic factors and lifestyle related factors were described with percentages. Initially, the proportions were estimated for cases and controls by performing univariate analysis. A probability value less than 0.05 was taken as the level of significance. An unadjusted odds ratio (OR) with a 95% confidence interval was calculated to assess the strength of each variable acting as a risk factor for an upper limb fracture. A variable which had an OR of more than one was considered to be a significant risk factor for upper limb fractures. A variable with an OR of less than one was considered as a protective factor, and if the OR was equal to one, that factor was not considered as a risk factor for upper limb fractures. The factors related to social habits namely smoking and alcohol consumption, pre-disposing factors namely episodes of fasting and previous history of epilepsy and food habits namely consumption of meat, fish and milk were excluded from bivariate analysis since these factors were not significant. Bivariate anlysis was performed to carryout logistic regression (LR) using backward LR method. The variables which had less than 10 in the case or control group, variables with large confidence interval in the univariate analysis with an upper limit of more than 30 and variables which were strongly corelated with other variables were also excluded. The dependent variables used in LR analysis were categorized as the presence of a fracture or the absence of a fracture. The presence of a fracture was coded as = 1 and the absence of a fracture was coded as = 0. LR was based on two assumptions:adequate sample size namely at least 10 cases per independent variable tested and the absence of multicollinearity between predictor variables. The variables that showed a significant association with having an upper limb fracture at a significance level of 0.05 were taken as independent variables. Variables retained in the logistic regression model were identified as determinants for upper limb fractures among adolescents in the district of Colombo adjusted for confounding factors. Goodness of fit of the model was assessed by the overall percentage of the prediction of having a fracture, the Chi squared test, Hosmer and Lemeshow test, Omnibus test, Cox and Snell Square test and Negelkerke R^2^ tests.

## Results

The study sample consisted of 450 cases and 450 controls. The response rate of the sample was 99.6%. The mean age of the cases was 13.62 years (SD = 2.8) with a probability of less than 0.001 (*p* < 0.001) and the mean age of control was 12.75 years (SD = 2.7) with a probability of less than 0.001 (*p* < 0.001). The standardized skewness for age in the study was 0.58 and the standardized Kurtosis was 0.67.

The basic characteristics of the cases and controls are shown in Table 2

**Table 2:** Distribution of demographic and socio-economic characteristics of cases and controls

Characteristic	Disease condition			
	**Cases (*N* = 450)**		**Controls (*N*** **= 450)**	
	No:	%	No:	%
**Sex**				
Male	371	82.4	246	54.7
Female	79	17.6	204	45.3
**Age**				
10–13	299	66.4	233	51.8
14–15	78	17.4	88	19.6
16–19	73	16.2	129	28.6
**Ethnicity**				
Sinhalese	330	73.3	398	88.4
Muslim	80	17.8	30	6.7
Tamil	39	8.7	20	4.5
Burger	1	0.2	2	0.4
**Religion**				
Buddhist	286	63.5	375	83.3
Catholic/ Christianity	64	14.2	45	10
Hindu	21	4.7	12	2.7
Islam	79	17.6	18	4
**Monthly income**				
Rs 10,000 -15,000	32	7.1	92	20.4
Rs 15,001- Rs 30,000	127	28.2	163	36.2
Rs 30,001- Rs 45,000	151	33.6	114	25.3
Rs 45,001- Rs 60,000	83	18.4	42	9.3
> Rs 60,000	53	11.8	28	6.2
Not known	4	0.9	11	2.4
**Social class**^a^				
Class I	38	8.2	17	3.8
Class II	44	9.5	19	4.2
Class III	202	43.5	98	21.8
Class IV	130	28.0	241	53.6
Class V	36	10.8	75	16.6
**Standard of Living**^b^				
High	139	30.0	110	24.4
Medium	280	60.3	247	54.9
Low	31	6.7	93	20.7

^a^Classification based on father’s occupation

^b^Classification based on validated instrument

Age and gender were assumed as predictors for upper limb fractures according to previous literature [11]. This study revealed that the risk of having a fracture increased gradually from the age of 12 to the age of 19 in males and vice versa in females. The univariate analysis of current study revealed that the age difference among adolescents was significant, indicating a two-fold risk of having an upper limb fracture among ages between 10 to 14 years (OR = 2.02; 95% CI = 1.5,2.7; *p* < 0.001). The sex difference of the participants was also significant with male sex having a higher risk for upper limb fracture (OR = 3.89; 95% CI = 2.87, 5.29; *p* < 0.001). The participants were categorized into two groups to perform univariate analysis to assess their social class and standard of living. The difference in standard of living was significant and there was a threefold risk of having upper limb fracture among participants belonged to high standard of living (OR = 3.52,95% CI = 2.3,5.4; *p* < 0.001). There was a twofold risk of having upper limb fracture “(OR = 2.58, 95% CI = 1.7;3.9, *p* < 0.001)” among participants who belonged to high social class status (social class I and II) and this difference was significant (Table 3).

**Table 3:** Risk of upper limb fractures associated with demographic and socioeconomic characteristics of adolescents

Characteristic	Disease status				OR	95% CI	Significance
	**Cases (*N*** **= 450)**		**Controls (*N*** **= 450)**				
	**No**	**%**	**No**	**%**			
**Age**							
10 to 14 years	351	78.2	284	64.0	2.02	1.50–2.71	χ^2^ = 21.94
> 14 to 19 years	98	21.8	166	36.0	1.0		*p* < 0.001
**Sex**							
Males	371	82.4	246	54.7	3.89	2.87–5.29	χ^2^ = 80.54
Females	79	17.6	204	45.3	1.0		*P* < 0.001
**Ethnicity**							
Sinhalese	330	73.3	398	88.4	2.78	1.95–3.98	χ^2^ = 33.2
Non -Sinhalese	120	26.7	52	11.6	1.0		*P* < 0.001
**Religion**							
Buddhists	286	63.6	375	83.3	2.8	2.09–3.92	χ^2^ = 45.13
Non-Buddhists	164	36.4	75	16.7	1.0		P < 0.001
**Parents employed**							
Employed	432	96.0	442	98.7	3.1	1.21–7.82	χ ^2^ = 6.14
Not employed	18	4.0	6	1.3	1.0		*p* = 0.021
**No: of siblings**							
One sibling	187	41.6	143	31.8	0.66	0.49–0.86	χ ^2^ = 9.26
More than one	263	58.4	307	68.2	1.0		*p* = 0.003
**Social class**							
High (Social class I &II	86	18.4	36	8.0	2.58	1.70–3.92	χ ^2^ = 21.01
Low (Social class III, IV & V)	364	81.6	414	92.0	1.0		*p* < 0.001
**Monthly family income**							
LKR^a^ 30,000 or less	3	0.7	24	5.5	0.12	0.35–0.39	χ ^2^ = 1.05^**α**^
More than LKR^a^ 30,000	447	99.3	426	94.5	1.0		*p* < 0.001
**Standard of living**							
High	419	93.1	357	79.3	3.52	2.29–5.41	χ ^2^ = 35.95
Low	31	6.9	93	20.7	1.0		*p* < 0.001

^a^Sri Lankan Rupees

The difference in engaged with sports or physical exercise was significant in cases and controls which showed a nine-fold risk of having upper limb fracture who had engaged with heavy intensity sports or physical exercise (OR = 9.36; 95% CI; 3.31, 26.47, *p* < 0.001). According to the study, there was a risk of having upper limb fracture among participants who were watching television on weekdays (OR = 1.95; 95% CI; 1.18, 3.22, *p* = 0.009) and playing video games or computer games on weekends (OR = 2.35; 95% CI; 1.7, 3.24, *p* < 0.001) Other variables that have significant OR are given in Table 4**.**

**Table 4:** Risk of upper limb fractures associated with lifestyle related factors

Characteristic	Disease status				OR	95% CI	Significance
	**Cases (*N*** **= 450)**		**Controls (*N*** **= 450)**				
	**No**	**%**	**No**	**%**			
**Sports/Physical exercise**^a^ (Mild/Moderate)							
Yes	226	47.4	194	57.1	1.48	1.11–1.96	χ^2^ = 7.19
No	229	52.6	146	42.9	1.0		p = 0.007
**Sports/Physical exercise**^a^ (Heavy)							
Yes	44	1.0	38	8.4	9.36	3.31–26.47	χ^2^ = 25.76
No	406	99.0	412	91.6	1.0		*p* < 0.001
**Sports related activity**^a^							
Yes	257	73.4	215	91.5	3.89	2.28–6.65	χ^2^ = 27.48
No	193	26.6	20	8.5	1.0		*P* < 0.001
**Leisure activities**							
Yes	316	87.8	290	95.7	3.1	1.64–5.88	χ^2^ = 13.17
No	44	12.2	13	4.3	1.0		*p* < 0.001
**Tuition/extra classes**							
Yes	201	56.9	221	67.2	1.82	1.18–2.78	χ^2^ = 7.56
No	152	43.1	108	32.8	1.0		*p* = 0.007
**Watching television**							
Yes	385	87.5	328	93.2	1.95	1.18–3.22	χ ^2^ = 7.03
No	55	12.5	24	6.8	1.0		*p* = 0.009
**Playing video/ computer games**							
Yes	93	21.4	124	39.0	2.35	1.7–3.24	χ ^2^ = 27.78
No	342	78.6	194	61.0	1.0		*p* < 0.001

^a^Classification based on guideline developed by Ministry of Sports, Sri Lanka

According to these results, the risk of having upper limb fractures among adolescents in Sri Lanka are age between 10 to 14 years, being a male adolescent, being a Sinhalese, being a Buddhist, Parent is employed, having a high standard of living index, belong to high social class category, mild to moderate intensity physical or sports activity, heavy intensity physical or sports activity, watching television, playing video or computer games and attending extra classes or tuition classes after school hours. Bivariate analysis was carried out with LR analysis and adjusted Odds Ratios (aOR) to identify individual risk for upper limb fractures adjusted for all confounders (Table 5).

**Table 5:** Adjusted Odds ratios for having risk factors for the variables with effect modification

Predictor variable	Adjusted OR (aOR)	Significance (p value)	95% CI for Exp (β)	
			**Lower**	**Upper**
**Sample size (n) > 10 cases per variable**				
High standard of living score	0.03	0.01	0.002	0.474
Religion (Being a Buddhist)	0.02	0.00	0.00	0.09
Siblings in the family	11.62	0.03	6.95	41.29
Attend tuition/extra classes	2.51	0.04	0.68	0.93
Playing video/computer games	0.19	0.04	0.039	0.91
Watching television	0.057	0.02	0.009	0.373

Following adjusting for confounders, the determinants for upper limb fractures were having siblings in the family (aOR = 11.62, 95% CI: 6.95, 41.29, *p* = 0.03) and attend extra classes after school hours (aOR = 2.51, 95%CI: 0.68–0.93, *p* = 0.04), high standard of living (aOR = 0.03, 95% CI:0.002, 0.474, *p* = 0.01), being a Buddhist (aOR = 0.02, 95% CI: 0.00, 0.09, *p* < 0.001) play video or computer games (aOR = 0.19, 95% CI: 0.039–0.91, *p* = 0.04), and watch television (aOR = 0.06, 95%CI:0.009, 0.373, *p* = 0.02) as shown in Table 5**.**

The final LR model was able to classify the cases from controls with 93.8% accuracy, compared to 75% without any of the independent variables used in the model. The Cox and Snell R square and Nagelkerke R square test results, 66% to 88.3% of the variability in the dependent variable is explained by the independent variables in the model.

The results of Hosmer and Lemeshow Goodness of Fit test were namely Chi-square (x^2^) test value = 18.1; df = 8: *p* = 0.02. The sensitivity of the model was 92.4% and the specificity was 87.6%. The positive predictive value was 92.9% while the negative predictive value of the model was 96.9%. Two significant effect modifications between being a Buddhist and low standard of living (*p* < 0.001) and having one sibling and attending extra classes (*p* = 0.01) were observed. Accordingly, being a Buddhist with low standard of living score had six-fold risk (OR = 6.35) of having an upper limb fracture than adolescent with high standard of living score. For an individual with one sibling with ever attended extra classes had two-fold risk (OR = 2.48) of having an upper limb fracture compared to those who did not attend extra classes.

Altogether, 16 factors were considered for the analysis and 15 variables were significant and six significant factors were retained in the model.

## Discussion

The Indoor Morbidity and Mortality Records (IMMR) of Sri Lanka revealed that the highest number of injuries was reported from the district of Colombo and fractures were common among adolescents [26]. Therefore, this study was carried out in the district of Colombo to determine risk factors for upper limb fractures among adolescents in Sri Lanka.

Previous literature revealed that the adolescents from low-income families have a higher risk of sustaining a fracture [27]. The findings of the current study showed that high standard of living is negatively associated with having an upper limb fracture (aOR = 0.03, 95% CI = 0.02,0.47, *p* = 0.01). A similar study carried out in Scotland described that low socioeconomic status was a significant risk factor for fractures among adolescents (*p* < 0.001) [8].

The findings of GSHS survey revealed that one fifth of adolescents were not physically active for at least 60 min per day and 37.3% of the students were not engaged in any activity and they preferred to sit for three or more hours per day in Sri Lanka [19]. It is evident that adolescents lead a sedentary lifestyle with lack of physical activity. This has led to increase in non-communicable diseases globally. The findings of the present study portrayed similar picture with regard to sustain upper limb fractures since there was a two-fold risk of having upper limb fractures associated with playing video games or computer games (OR = 2.35; 95% CI; 1.7, 3.24) and watching televisions (OR = 1.95; 95% CI; 1.18, 3.22). These findings were supported by the findings of Deoiong & Graeme where the time spent on television, computer, and watching videos in both sexes had a significant relationship with a 1.6-fold risk of having wrist and forearm fractures (OR = 1.6; 95% CI; 1.1, 2.2) [13]. Heads of the schools need to pay more attention to allocate more time for outdoor activities in this age group. The present education system in Sri Lanka also encourages them to have a sedentary lifestyle due to high competition in Advanced Level examination in the country. They attend extra classes after school hours due to this competition and sit at one place for long duration. As such attending extra classes was a significant risk factor to have upper limb fractures among adolescents in Sri Lanka (aOR = 2.51, 95% CI: 0.68, 0.93, *p* = 0.04). The main risk of having upper limb fracture in this study was related to the modifiable risk factors such as socioeconomic background and lifestyle related factors of these adolescents. The risk factors related to lifestyle can be prevented by creating awareness of lifestyle modifications. This is an important finding for the planners of preventive programmes in the country, where the sedentary lifestyle among adolescents must be discouraged to prevent from risk of having upper limb fractures as well as to prevent from other non-communicable diseases. Deoiong & Graeme further revealed that adolescents were disturbed behaviorally and psychosocially by watching television or playing computer and video games [13]. Previous literature had revealed that television viewing during early adolescent age was significantly associated with aggressive behavior (aOR = 1.46; CI; 1.05–2.60) [28]. There was a nine-fold risk of upper limb fracture in those who were engaged in heavy intensity sports or physical activities (OR = 9.36; 95% CI: 3.31, 26.47). The findings were compatible with a study done by Clerk et al. [14] where a two-fold risk of upper limb fracture was observed with vigorous physical activity in the United Kingdom (OR = 2.06; 95% CI: 1.21, 1.76). However, very intense physical exercises should also be discouraged during early adolescence according to the guidelines developed by the Ministry of Sports. These guidelines should be disseminated and implemented in schools and youth clubs. Thus, in general, the promotion of sports and physical exercise programmes in the school curriculum is recommended to reduce the sedentary life styles of adolescents. 

Of the potential risk factors, being limited to sedentary recreational activities namely watching television or playing computer or video games, attending extra classes and the adolescent’s standard of living index were determinants of upper limb fractures among adolescents in Sri Lanka which were modifiable and can be prevented as risk factors. The present study found that sedentary lifestyle may lead to musculoskeletal diseases even at a young age. Therefore, there is a need to strengthen the awareness programmes and preventive activities to combat the risk factors for unintentional injuries among adolescent population in Sri Lanka. The Ministry of Health can strengthen its home safety programmes by disseminating home safety checklists to implement safe home environments by increasing awareness regarding home safety in the community. Further, the child injury prevention booklets developed by the Ministry of Health, Sri Lanka can be utilized by public health staff to increase public awareness on child safety. This will be a more cost-effective preventive method to deal with adolescent and child injuries by reducing falls and other mechanisms of injuries among children and adolescents. Educating the parents on the risk-taking behavior of their children is another important preventive measure. The public health staff can be utilized for awareness programmes as such Sri Lanka is blessed with dedicated public health staff at district level. In addition, there is a National Injury Surveillance system at district level to take further action. The Non-Communicable Disease unit (NCD) for acute NCD has already taken steps for injury prevention such as awareness programmes, child safety programmes and prehospital care programmes to empower the community through health promotion. The Ministry of Health in Sri Lanka has also identified a healthy school concept with a hazard free school environment for the future generation. Further, the GSHS survey recommended the regular assessment of hazards in school environment for the safety of school children [19].

The findings of this research were already disseminated to policy makers to implement preventive strategies for adolescent injuries. 

### Strengths and limitations

There was no single study available in Sri Lanka, which addressed the potential risk factors for upper limb fractures among the adolescent population. All major hospitals in the district of Colombo were included in the current study. Colombo is the highly commercialized and most populated district in Sri Lanka. It was possible to conduct the study in this way, as many adolescents with upper limb fractures attended these hospitals. Therefore, the authors are aware that the findings cannot be generalized to other districts. Although there is a possibility of the risk magnitude to be differed for each district in Sri Lanka, the risk factor profile can be generalized to the country.

The cases and controls were not matched as the magnitude of the effect of potential risk factors such as age and sex had already been assessed as risk factors in the present study. The previous literature covering the local context reported results for unmatched case control studies for similar risk factors [16, 17]. However, confounders were controlled by performing logistic regression analysis. Recall bias and information bias were minimized in the current study by recruiting new cases within one week following an injury [29]. The ideal control group for this type of study is apparently healthy adolescents from the community who did not have previous upper limb fracture or presenting with a fracture. However, selection bias cannot be fully excluded since the source of controls were not from hospital setting as cases. The present study adopted several measures to minimize the selection bias since it is a specific source of sampling error in the case control design [29]. The current study fulfilled this requirement by recruiting apparently healthy adolescents from the community.

## Conclusions

'This study emphasizes the importance of early detection of modifiable risk factors that can increase the risk of upper limb fractures among adolescents. Upper limb fractures can have a major impact on quality of life of adolescents and young adults. Results of this study had been shared with the relevant authorities to identify, plan and implement preventive measures related to adolescent injuries in Sri Lanka. Further, the findings will also be disseminated via communications in scientific forums and publications in scientific journals, both national and international.

Future researchers need to address health issues among adolescents, especially those related to unintentional injuries, as this is still a neglected public health problem in Sri Lanka. Future studies could evaluate such strategies as a means to prevent upper limb fractures and associated injuries among adolescents in Sri Lanka.

## Data Availability

The Datasets used during the current study are available from the corresponding author on reasonable request. All methods were carried out in accordance with relevant guidelines and regulations.

## Abbreviations

WHO: World Health Organization
UNICEF: United Nations International Children’s Emergency Fund
OR: Odds Ratio
CI: Confidence Interval
SD: Standard Deviation
BMD: Bone Mineral Density
ASU: Accident Service Unit
PCU: Primary Care Unit
PHM: Public Health Midwife
LR: Logistic Regression
GSHS: Global School Health Survey
SPSS: Statistical Package for the Social Science
